# Effects of repetitive transcranial magnetic stimulation on upper extremity motor function in stroke survivors: study protocol of a randomized sham-controlled trial

**DOI:** 10.3389/fneur.2025.1669862

**Published:** 2026-01-02

**Authors:** Tao Sun, Qian Yu

**Affiliations:** 1 Department of Rehabilitation, Affiliated Hospital of Southwest Medical University, Luzhou, China; 2 Department of Rehabilitation Medicine, Sichuan Academy of Medical Sciences, Sichuan Provincial People’s Hospital, Chengdu, China

**Keywords:** high-frequency repetitive transcranial magnetic stimulation, dorsolateral premotor cortex on the unaffected side, stroke, functional magnetic resonance, sham control, motor evoked potentials

## Abstract

**Background:**

Stroke is a group of diseases with neurological deficits caused by cerebrovascular lesions. Despite standard treatment, a large number of patients are left with significant upper extremity motor dysfunction. Therefore, improving upper limb motor function and promoting neurological recovery in stroke patients has become a core challenge in the field of neurorehabilitation.

**Methods and analysis:**

This study will be designed as a randomized, sham controlled clinical trial. Thirty-six patients with upper limb motor dysfunction after stroke will be included, and their Fugl-Meyer Upper Extremity Motor Function Rating Scale (FMA-UE) score will be ≤ 26 points. Subjects will be randomly assigned to the following three groups (*n* = 12/group): HF-rTMS: Treated with high-frequency (5 Hz) repetitive transcranial magnetic stimulation (rTMS) on the dorsal premotor cortex (PMd) on the contralesional side. Low-frequency group (LF-rTMS): Treated with low-frequency (1 Hz) rTMS on the primary motor cortex (M1) on the contralesional side. Sham control group: The stimulation target and parameter settings will be the same as those of the high-frequency group, but the coil will be placed perpendicular to the scalp plane. The intervention regimen will be rTMS treatment once daily, five times a week for 3 weeks. Outcome measures: Main outcome measures: Upper limb motor function, assessed by FMA-UE. Secondary outcomes: Barthel Index (BI), National Institutes of Health Stroke Scale (NIHSS). Exploratory indicators: fMRI data and motor evoked potential (MEP) parameters. All assessment data will be collected before and after the intervention.

**Communication and ethics:**

This study has been reviewed and approved by the Basic and Clinical Research Ethics Committee of Sichuan Provincial People’s Hospital (Lun Shen (Yan) No. 353–1 of 2025). The results of this research will be disseminated through the network of professionals and the general public, and peer-reviewed scientific papers will be published and presented at relevant conferences.

**Registration:**

This trial has been registered with the Chinese Clinical Trials Registry with registration number ChiCTR2500105502.

## Introduction

Stroke is a group of neurological dysfunction diseases caused by cerebrovascular lesions. Clinically, such patients are often accompanied by varying degrees of motor and sensory dysfunction. The disease is characterized by high morbidity, disability and mortality rates, and has become the leading cause of disability in China (1, 2). Studies have shown that even with early intervention and standardized treatment, 55–75% of stroke patients still have persistent upper limb motor dysfunction (3, 4). Such dysfunction has a long recovery cycle and poor curative effect, which is strongly correlated with the significant decline in activities of daily living (ADL) and the deterioration of quality of life after stroke (5–8), and brings heavy economic burden to the social medical system and family care. Epidemiological data show that about 50% of stroke survivors have severe upper limb motor dysfunction (9), which is characterized by the near loss of voluntary mobility of shoulder, elbow, wrist and hand joints, resulting in the inability to complete basic daily activities (10). Current treatment strategies for patients with severe dysfunction are limited and clinical response rates are not ideal. Therefore, it is still a scientific problem that needs to be broken through in the field of neurorehabilitation to explore effective intervention methods to improve severe upper limb motor dysfunction after stroke, promote the mechanism of neural remodeling, and maximize the recovery of patients’ ADL ability.

Repetitive Transcranial Magnetic Stimulation (rTMS), as a non-invasive neuromodulation technology, evokes induced currents in cortical neurons through time-varying magnetic fields based on the principle of electromagnetic induction, and then regulates target cortical excitability, neuroplasticity and neurometabolism-electrophysiological activities (11–13). Current clinical practice mainly employs two modes: high-frequency stimulation (> 1 Hz) enhances cortical excitability and low-frequency stimulation (≤ 1 Hz) inhibits cortical excitability (14). This technique has been widely used in the treatment of neuropsychiatric diseases such as Parkinson’s disease and depression, and has been confirmed to have significant clinical efficacy (15). In the field of stroke rehabilitation, mainstream intervention strategies follow the Interhemispheric Competition Model (16): high-frequency rTMS targets the primary motor cortex of the ipsilesional side (iM1) to enhance the excitability of the damaged hemisphere; low-frequency rTMS acts on the primary motor cortex of the contralesional side (cM1) to inhibit the hyperexcitability of the contralesional hemisphere. However, this model has limitations in patients with severe injury to the Corticospinal Tract (CST) (17–19). With significant loss of corticospinal connectivity on the affected side (20), conventional interventions based on the competitive model are difficult to induce effective functional recovery. In response to this problem, Di Pino et al. proposed the Bimodal Balance-Recovery Model (BBRM) (12), pointing out that when the integrity of CST on the ipsilesional side is severely damaged, the contralateral hemisphere compensatory mechanism may dominate the reconstruction of motor function. Exciting the contralateral cortex may be a better strategy. In addition, most repetitive transcranial magnetic stimulation (rTMS) experiments in stroke rehabilitation studies focus only on the M1 area. When the corticospinal tract (CST) is severely damaged, the pattern of brain reorganization may not only be related to the M1 region, but also involve secondary motor areas closely connected to M1. Existing studies have shown that the dorsal premotor cortex (PMd) is involved in the coordination and control of complex hand movements (21). Therefore, for patients with severe brain injury, the contralateral dorsal premotor cortex (cPMd) could be a more valuable alternative, given its higher survival probability and richer descending projection network (22).

In this randomized sham-controlled trial, functional Magnetic Resonance Imaging (fMRI) and Motor Evoked Potentials (MEP) were integrated in addition to the standardized upper limb motor function scale. The main objectives of this experiment are as follows: 1. To analyze the dynamic changes in brain network structure and functional connectivity after intervention. 2. To establish a correlation model between clinical functional improvement and neuroimaging/electrophysiological indicators. 3. Explain the neural remodeling mechanism of upper limb functional rehabilitation after stroke (23). Core scientific hypothesis: High-frequency rTMS targeting the contralateral dorsal Premotor Cortex (cPMd) can significantly promote the recovery of upper limb motor function in patients with severe stroke. This study will provide new evidence for the mechanism of rTMS regulating neural compensation after stroke, and promote the development of precise neurorehabilitation strategies.

## Methods

### Objectives

To explore whether the high-frequency rTMS scheme of cPMd can more effectively compensate for the instability of its therapeutic effect than the low-frequency repetitive transcranial magnetism (rTMS) scheme of cM1.

### Study design

**Figure 1:**
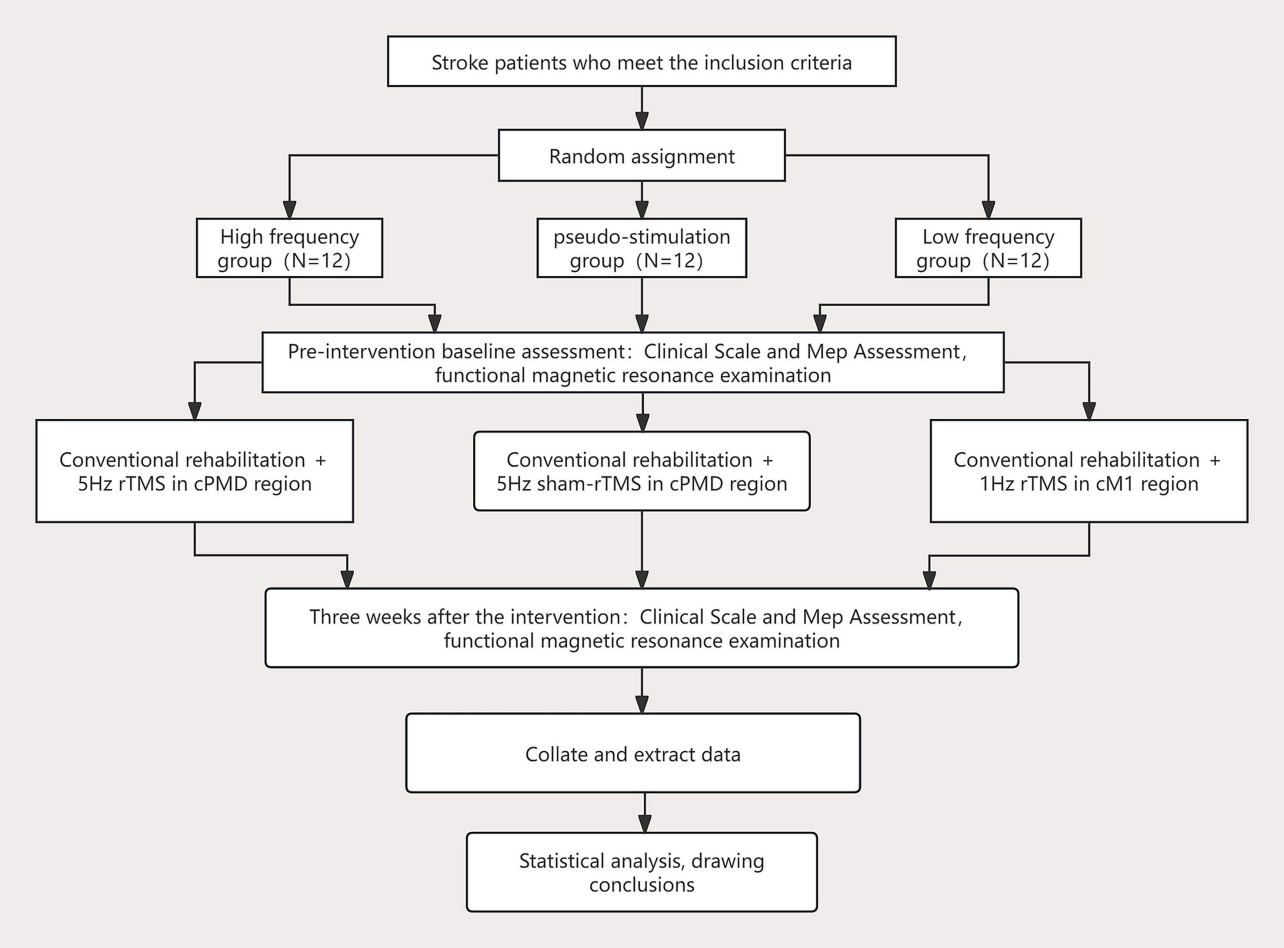
Flow chart showing the study design. cM1, contralesional primary motor cortex; rTMS, repetitive transcranial magnetic stimulation; cPMd, contralesional dorsal premotor cortex; MEP, Motor Evoked Potentials.

This study is a randomized sham-controlled clinical trial divided into high-frequency, low-frequency and sham-control groups. This study will be carried out in the Department of Rehabilitation Medicine of Sichuan Provincial People’s Hospital. Potential patients will be provided with detailed project information as well as full interpretation of informed consent. In order to improve patient compliance and reduce dropout rates, we will fully communicate before the trial to ensure that patients fully understand the significance of the study and voluntarily sign an informed consent form to participate. Subjects who met the inclusion criteria will be randomly assigned to three groups at a 1: 1: 1 ratio (n = 12 per group): high-frequency intervention group (5 Hz rTMS in the contralateral PMd region); Low-frequency intervention group (1 Hz rTMS in the ipsilesional M1 region); Sham stimulation control group (parameters were the same as high frequency group, coils were placed vertically). This study protocol has been reviewed and approved by the Basic and Clinical Research Ethics Committee of Sichuan Provincial People’s Hospital (Lun Shen (Yan) No. 353–1 of 2025), and has been registered with the China Clinical Trial Registry (ChiCTR) (registration number ChiCTR2500105502). Figure 1 shows the experimental flow chart.

### Participants and eligibility

#### Enrollment criteria

The following are the inclusion criteria: (1) meet the diagnostic criteria for stroke: according to the “Guidelines for the Diagnosis and Treatment of Cerebral Hemorrhage in China (2019)” or “Guidelines for the Diagnosis and Treatment of Acute Ischemic Stroke in China (2018),” the diagnosis is confirmed by head CT/MRI imaging; (2)characteristics of stroke attack: initial unilateral onset, or no sequelae of neurological deficit left by previous stroke attacks; (3) vital signs and consciousness state: Vital signs are stable for ≥ 24 h, Glasgow Coma Scale (GCS) score = 15 points;(4) age range: 18–75 years old; (5) upper limb motor deficit: baseline Fugl-Meyer Upper Limb Motor Function Rating Scale (FMA-UE) score ≤ 26 points (suggesting severe motor impairment) (18); (6) informed consent: Voluntary written informed consent; (7) Recruiting patients within 1 week to 6 months after stroke.

#### Exclusion criteria

The following are the exclusion criteria: (1) severe functional impairment: significant cognitive impairment (MMSE < 24), visual field impairment, hearing impairment or aphasia (confirmed by Boston Diagnostic Aphasia Test); (2) contraindications of rTMS: acute craniocerebral trauma or cerebral hemorrhage, history of epilepsy or epileptiform discharge detected in EEG; (3) unstable general condition: uncontrolled hypertension (SBP > 180 mmHg), severe heart, liver and kidney failure (NYHA grade III-IV), active malignant tumor; (4) motor system complications: modified Ashworth spasticity scale score ≥ 3 or rest tremor (UPDRS-III tremor item ≥ 2 points); (5) MRI/TMS contraindications: implantation of electronic devices (cardiac pacemakers, drug pumps, etc.), non-titanium alloy intracranial aneurysm clips, skull metal implants; (6) unable to cooperate with the examination: claustrophobia (clinical diagnosis) or fMRI tolerance failure caused by movement disorder; (7) pregnant or lactating women (negative urine pregnancy test before enrollment in women of childbearing age).

#### Sample size calculations

In this study, in order to evaluate the effect of three different therapies on upper limb motor function rehabilitation in patients with severe stroke hemiplegia, the upper limb part of the Fugl-Meyer rating scale (FMA-UE) is used as the main efficacy index. The subjects are divided into high frequency group, low frequency group and sham control group according to the principle of random control. According to the relevant literature and pre-experimental results, the average FMA-UE of each group was 11.4, 4.7 and 2.8, and the standard deviation was 1.4, 0.9 and 1.3, respectively. When the *α* of the bilateral test value is 0.05 and the power of the power is 0.9, according to the PASS15 software “Means—One WayDesigns (ANOVA)—ANOVA-Test—One-Way Analysis of Varianc F-Tests,” it is calculated that at least 9 cases need to be enrolled in each group, and considering the shedding situation, according to the calculation of 20% dropout rate, at least 12 cases are needed in each group, A total of at least 36 participants are required in the three groups (24).

#### Randomization and blinding

Based on the sample size calculation, this study will enroll a total of 36 eligible subjects. Patients will be numbered sequentially according to their enrollment time and randomly assigned to one of three groups in a 1:1:1 ratio using a random number table. The study personnel responsible will number the patients and perform the randomization. Each numbered treatment protocol will be placed individually into an opaque, sealed envelope. Therapists will open the envelopes in numerical order and administer the treatment according to the corresponding protocol. The group codes will not be revealed until all analyses are completed. The sham stimulation group will use a mock device identical in appearance to the real equipment to replicate the look and acoustic noise of the active coil. The TMS operators will not be involved in trial design, assessment, or data analysis. Except for discussing discomfort or adverse effects, they will be prohibited from communicating any trial details with the subjects. All clinical assessors, behavioral therapists, data analysts, and other research staff will remain blinded to the group assignments (25).

#### Intervention

##### Treatment equipment

YRDCCY-I repetitive transcranial magnetic instrument (YRDCCY Medical Technology Co., Ltd.), equipped with 70 mm double-ring “8” coil (peak magnetic field intensity ≥ 2.2 T).

##### Target positioning process

When locating the target, the operator first determines the position of the CZ point on the scalp surface of the patient. According to the EEG 10–20 positioning system: Step 1: Measure the total length from the nasal root point to the external occipital carina point, record this length as L, start from the nasal root, move 50% of the L distance backward along this line, Cz1 point is at the midpoint of this midline. Step 2: Measure the total length of the left pre-auricular point passing through the top of the head to the right pre-auricular point, record this length as M, start from the left pre-auricular point, and move 50% M distance to the right along this line. The Cz1 point in the first step coincides or is extremely close, and this final intersection point is the Cz point. The CZ point corresponds to the anterior gyrus of the cerebral cortex about 5 cm lateral to both sides, and the plane of the midpoint of the coil is tangent to the surface of the scalp here, looking for the position that can trigger the maximum MEP amplitude of the contralateral abductor pollicis brevis (26), that is, the contralateral primary motor cortex (cM1), which is used as the target of rTMS in the low-frequency group. About 2.5 cm anterior to cM1 is cPMd (27), which is used as a target for rTMS in the high frequency group.

##### Treatment parameters

**Figure 2:**
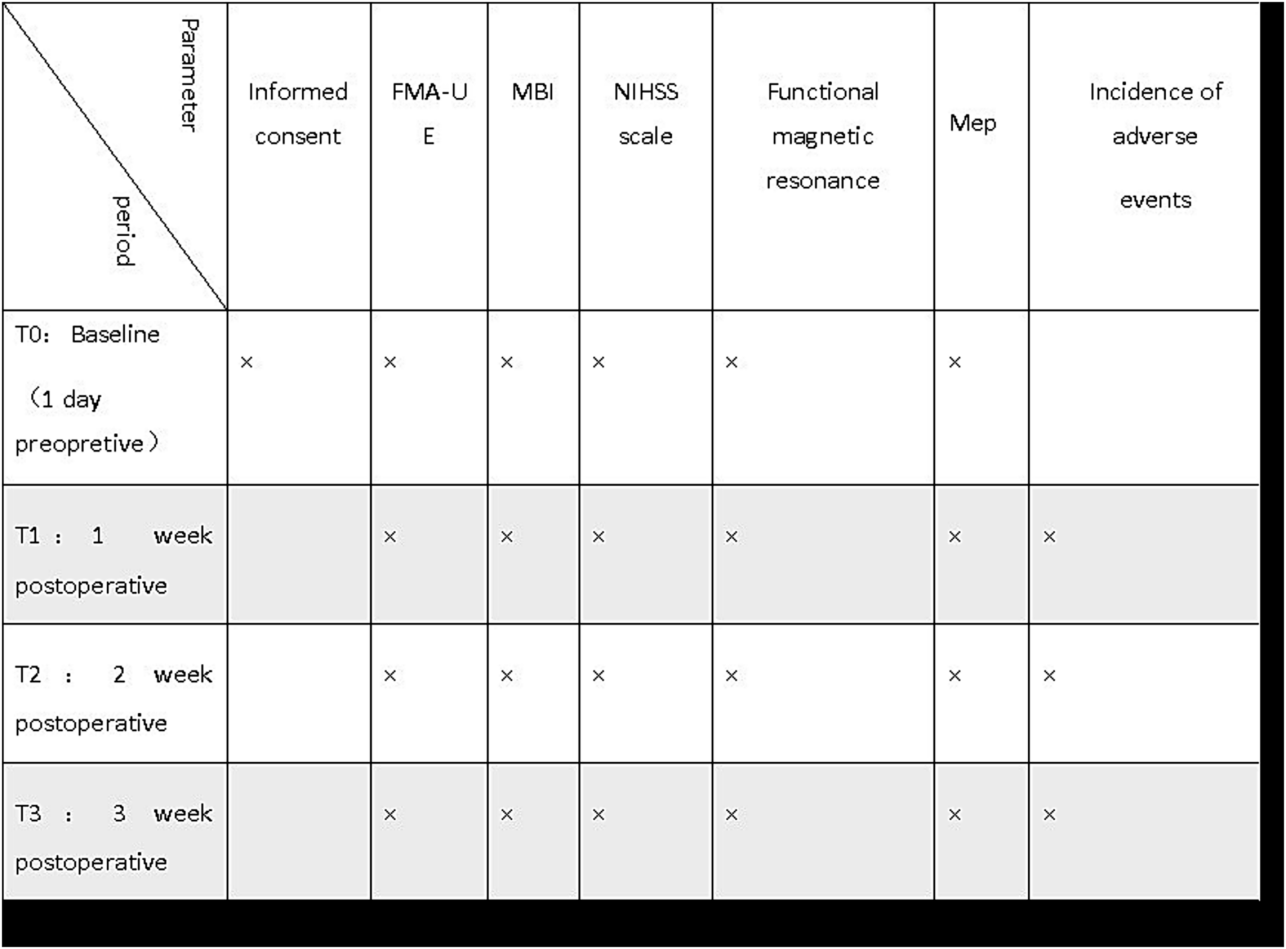
Schedule of participant enrolment and assessments. FMA-UE, Fugl-Meyer assessment of upper extremity; MBI, Modified Barthel Index; MEP, Motor Evoked Potentials; NIHSS Scale, National Institutes of Health Stroke Scale.

All three groups will be treated with rTMS in the same time with conventional drug therapy and rehabilitation training. During treatment, the patients will be placed in the lying position, and the “8” shaped coil will be placed near the tangent line of the scalp. Parameters of the low frequency group: The site is the M1 area of the cerebral cortex on the contralesional side, using 1 Hz, the intensity is 90% of the resting motor threshold, each sequence is 20s, 20 sequences will be repeated, intermittent for 1 s, and the total number of pulses is 1,200, once a day, 5 times a week, the course of treatment is 3 weeks. Parameters of high frequency group: PMd in the healthy cerebral cortex will be used as the target of high frequency group, at 5 Hz, the intensity is 90% of the resting motion threshold, each sequence is 6 s, 30 sequences will be repeated, intermittent for 6 s, the total number of pulses is 1,200, 5 times a week, and the course of treatment is 3 weeks. Parameters of the sham control group: The target and related parameters are the same as those of the high-frequency group, but the coil will be rotated 90 degrees to make it similar to the scalp of the participant and the discharge noise is similar to that of 5 Hz, so that the induced current in the brain will be minimized (28–30). The relevant time periods of the experiments are shown in Figure 2.

### Outcome assessment

#### Primary outcome measure

The FMA-UE (Functional Motor Assessment Scale-Upper Extremity) will be used to assess motor function in the paralyzed upper extremity. A total of 10 items, including reflex activity, flexor muscle movement, extensor muscle movement, activity with common movement, dissociative movement, hyperreflexia, wrist stability, wrist movement, finger movement, coordination, and speed, are scored on a total of 66 points, with higher scores indicating milder dyskinesia (31).

### Secondary outcome

#### Scale and electrophysiological assessment

1. Modified Barthel index (MBI): including 10 activities closely related to daily living, such as eating, bathing, grooming, and dressing, which are used to assess the ability of daily living activities, the higher the score, the higher the independence of the patient’s daily life, and the score greater than 60 indicates that the patient is likely to be able to complete the activities of daily living and maintain a normal standard of living (32).
2. National Institutes of Health Stroke Scale (NIHSS Scale): It includes a multi-faceted evaluation of the patient’s state of consciousness, language, sensory and motor function, which is used to evaluate the degree of neurological deficit, and the higher the score, the more serious the patient’s neurological impairment.
3. Motor evoked potential (MEP): Before and after treatment, the upper limb MEP is measured for the patient, first the upper limb on the unaffected side and then the upper limb on the affected side; The electrode is recorded by electrodes attached to the abductor pollicis brevis muscle, the reference electrode is attached to the tendon, connected to the ground wire, and transcranial magnetic single stimulation is performed in the functional area of the opposite hand of the tested limb to observe the waveform and the patient’s limb. The latency and amplitude are recorded.

#### fMRI scan

The acquisition will be carried out by the same professional and technical personnel in the magnetic resonance room of our hospital according to the prescribed acquisition sequence and parameters. Before the start of data collection, the contraindications of MRI examination will be checked again to see that the subjects do not have metal stents, pacemakers, etc., and the scanning steps will be introduced in detail, and the subjects will be instructed to lie flat on the scanning table in a resting and quiet state, and at the same time wear noise-canceling headphones to minimize the impact of the noise of the examination equipment on the collection results. During the scan, the subject will be asked to close his eyes and relax as much as possible, keep his head and body still, do not think or sleep, and start the scan after the subject is used to it. T1 structural image: the magnetization intensity will be used to prepare the gradient echo sequence, and the scanning parameters are as follows: time of repetition (TR) = 1900 ms, time of echo (TE) = 2.52 ms, field of view (FOV) = 250 mm × 250 mm, flipangle (FA) = 9°, matrix (Matrix) = 256 × 256, Thickness = 1.0 mm, Gap = 0 mm, voxel size = 1 × 1 × 1 mm^3^. rs-fMRI scanning: a gradient echo single excitation echo plane imaging is used, and the scanning parameters are: repetition time = 2000 ms, echo time = 13 ms, field of view = 192 mm × 192 mm, inversion angle = 90°, matrix = 64 × 64, layer thickness = 3.0 mm, layer spacing = 1 mm, voxel size = 3 × 3 × 3 mm^3^.

Data processing will be pre-processed with functional imaging data using resting-state functional magnetic resonance imaging software. Lesion size and fraction of anisotropy (FA) within the posterior internal capsule (PLIC) are determined by diffusion tensor imaging (DTI) to assess the integrity of the corpus callosum (CST). Anisotropy fraction (FA) values and mean anisotropy fraction asymmetry index (FAAI) are key indicators to assess bundle integrity (33, 34). DTI datasets will be processed using the Pipeline for Analyzing Brain Diffusion Images (PANDA) toolkit^1^ to obtain the average FA and FAAI values of patients from the whole group (35). The PANDA toolkit mainly includes the following steps: data preprocessing, diffusion tensor model fitting and index calculation, fiber tracing, brain structural network construction, and result output. Bilateral PLIC, Pontine and Anterior Gyrus will be selected as the target regions.

#### Data collection and management

In order to ensure the reproducibility, transparency and trust of the research, as well as to reduce data errors and biases, ensure data quality, and support accurate data analysis and reliable scientific conclusions, the study adheres to the following principles:

1. Ethics first: Ensure informed consent is obtained before data is collected, respecting privacy and cultural sensitivities. Comply with ethical review requirements to minimize potential risks to participants, the environment and society.
2. Equitable access and inclusion: By avoiding improper exclusion in the participant recruitment process and formulating inclusion and exclusion criteria based on scientific reasons, we can ensure that the data collection objects are representative, avoid systemic bias, unswervingly follow FAIR principles, and promote Universality and fairness of research results.
3. Quality first: Design a rigorous data collection plan, clarify the definition of variables and the collection process, and implement a real-time verification and cleaning mechanism to ensure the accuracy, completeness and consistency of data. Regular and rigorous monitoring of the trial will be carried out on the following aspects: division of work and training of researchers, file management, informed consent, protocol compliance, subject recruitment, intervention, statistical analysis, etc.

#### Data processing and analysis

We will use the Shapiro–Wilk test to assess whether the data are normally distributed. For data that conform to the normal distribution, it will be presented as means and standard deviations; for non-normally distributed data, it is presented as a median and interquartile range. For continuous data, one-way ANOVA will be used for between-group mean comparison; for categorical data, the chi-square test will be used for data analysis. The FMA-UE scale, BI score, NIHSS scale and imaging indicators [anisotropy score (FA) value and mean anisotropy score asymmetry index (FAAI)] will be analyzed by repeated measures ANOVA and multivariate ANOVA, respectively, in which the inter-group factors are high-frequency group, low-frequency group and sham stimulation group, and the intra-group factors are time (T0-T3), and multiple comparisons of LSD correction will be performed. The correlation between clinical scale data and imaging index data will be tested by Spearman or Pearson correlation tests. The level of statistical significance will be set at *p* < 0.05. In order to compare the therapeutic effects of different repetitive transcranial magnetic regimens, a two-factor mixed design analysis of variance including the inter-group factor “group” and the intra-group factor “time” will be used for clinical manifestations and electrophysiological changes. The DTI dataset will be processed via the PANDA toolkit to obtain the average FA and FAAI values for the entire population of patients.

## Discussion and conclusions

In recent years, many studies have confirmed that repetitive transcranial magnetism (rTMS) can effectively improve the motor function of stroke hemiplegic patients (36, 37). But there are also studies that have found negative results, and some patients even have functional degeneration (38, 39). These evidences suggest that standardized rTMS protocols based on the theory of bilateral primary motor cortex (M1) IHI imbalance have limitations in clinical practice (40). Patients with chronic stroke accompanied by severe motor impairments have widespread damage to the neural pathways in the affected hemisphere, resulting in the M1 region being unable to effectively promote motor function recovery, ultimately leading to the failure of repetitive transcranial magnetic stimulation (rTMS) therapy (41). Therefore, this study will focus on cPMd therapies based on the latest “bimodal equilibrium-recovery model” (12).

This study is the first to systematically evaluate the efficacy of high-frequency rTMS targeting the anterior dorsal motor cortex (cPMd) on the unaffected side on severe upper limb motor dysfunction after stroke. Based on the theoretical framework of the Bimodal Balance-Recovery Model (BBRM), we propose the following core mechanism hypothesis: when the corticospinal tract on the affected side is severely damaged, high-frequency stimulation of the unaffected side cPMd can significantly improve the recovery of motor function by enhancing transhemispheric compensatory nerve remodeling. This effect may involve: (1) reversal of inhibitory regulatory imbalance: regulation of pathological interhemispheric inhibition; (2) activation of compensatory pathways: promote synaptic plasticity of the unaffected hemisphere-spinal anterior horn network. The results of this study will provide key evidence for precision neurological rehabilitation: in the future, rTMS treatment plans need to stratify patients according to the time window of injury, lesion localization and CST integrity, so as to achieve individualized intervention. In addition, the cPMd high-frequency stimulation paradigm is expected to be extended to the neuromodulation of multi-domain dysfunctions such as aphasia and swallowing disorders after stroke.

The protocol has some limitations. First, the small sample size and single-center study design may affect the universality of the results. Second, due to clinical feasibility considerations, the timing of conventional treatment and repeated transcranial magnetic therapy is not strictly regulated, which may affect the interpretation of efficacy-although the randomization design may partially alleviate the time difference between groups. Future studies should control this variable more strictly. Third, this study will only evaluate the resting motion threshold of the contralateral hemisphere of the lesion before the intervention, and does not evaluate the bilateral hemispheres synchronously before and after the intervention, which will limit the verification of the theory of physiological effects of transcranial magnetic therapy and hemispheric imbalance. In addition, some subjects did not record the coordinate data of the hot zone of hand movement, which will lead to limitations in the comparison of differences between superior neural targets and hot zone targets.

## Footnotes

1
http://www.nitrc.org/projects/panda
